# β-Glucan and Dark Chocolate: A Randomized Crossover Study on Short-Term Satiety and Energy Intake

**DOI:** 10.3390/nu6093863

**Published:** 2014-09-23

**Authors:** Asli Akyol, Halil Dasgin, Aylin Ayaz, Zehra Buyuktuncer, H. Tanju Besler

**Affiliations:** 1 Department of Nutrition and Dietetics, Faculty of Health Sciences, Hacettepe University, Sihhiye, Ankara 06100, Turkey; E-Mails: baylin@hacettepe.edu.tr (A.A.); zbtuncer@hacettepe.edu.tr (Z.B.); htbf@hacettepe.edu.tr (H.T.B.); 2 Department of Nutrition and Dietetics, Faculty of Health Sciences, Kirikkale University, Yahsihan, Kirikkale 71450, Turkey; E-Mail: halil.dasgin@gmail.com

**Keywords:** β-glucan, dark chocolate, satiety

## Abstract

Aim: The aims of this study were to adapt a traditional recipe into a healthier form by adding 3 g of oat β-glucan, substituting milk chocolate to dark chocolate with 70% cocoa, and to examine the effect of these alterations on short-term satiety and energy intake. Materials and Methods: Study subjects (*n* = 25) were tested in a randomized, crossover design with four products closely matched for energy content. Four different versions of a traditional recipe including milk chocolate-control (CON), oat β-glucan (B-GLU), dark chocolate (DARK) or oat β-glucan and dark chocolate (B-GLU + DARK) were given to subjects on different test days. After subjects were asked to report visual analog scale (VAS) scores on sensory outcomes and related satiety for four hours *ad libitum*, lunch was served and energy intake of individuals was measured. Results: VAS scores indicated that none of the test foods exerted an improved effect on satiety feelings. However, energy intake of individuals during *ad libitum* lunch was significantly lower in dark chocolate groups (CON: 849.46 ± 47.45 kcal *versus* DARK: 677.69 ± 48.45 kcal and B-GLU + DARK: 691.08 ± 47.45 kcal, *p* = 0.014). Conclusion: The study demonstrated that substituting dark chocolate for milk chocolate is more effective in inducing satiety during subsequent food intake in healthy subjects.

## 1. Introduction

Addition of dietary fiber to energy-dense foods has been shown to be a successful strategy in development of healthy products [1,2,3,4]. Recent research has documented that soluble and viscous fiber in oat and barley slows gastric emptying and enhances the satiety feeling [5,6]. More specifically, oat fiber was shown to be effective in lowering serum cholesterol and blood pressure, reducing cardiovascular disease risk, as well as improving type 2 diabetes symptoms and antioxidant activity [7,8,9,10,11]. Oat fiber is a rich source of the water-soluble fiber β-glucan and the beneficial effects have largely been attributed mainly to β-glucan content of oat fiber [12]. In addition to disease-related influences, recent studies have also focused on the satiety effects of β-glucan with previous studies showing significant effects of β-glucan on enhancement of satiety feeling and decline in energy intake [13,14,15]. However, other studies indicated small or no effect of β-glucan on satiety, food and energy intake [16,17].

The dietary composition of chocolate has been considered unhealthy [18]. However, recent studies indicate that dark chocolate, which has a greater content of cocoa and lower content of sugar when compared to milk chocolate, is associated with a reduction in blood pressure, cardiovascular diseases, type 2 diabetes, fat deposition and mortality rates [19,20,21,22,23]. Sorensen and Astrup [24] have demonstrated the significant effect of dark chocolate on a greater satiety feeling, lower energy intake in prospective meal and reduced appetite for sugary snacks in comparison to milk chocolate consumption. These effects of dark chocolate were largely attributed to its higher cocoa content [24,25]. Therefore, replacement of milk chocolate with dark chocolate may be an easily achievable strategy in promoting satiety and reducing over-nutrition and subsequent nutrition-related disease risks.

A large body of evidence has shown the beneficial effects of functional foods on promoting health and reducing risk of certain diseases [26,27]. However, the efficiency and influence of the combined effects of these components has not been studied in detail [28]. In this context, the aim of the current study was to investigate the short-term effects of modified versions of a traditional recipe according to its dark chocolate and β-glucan content on appetite-related sensory parameters and prospective energy intake.

## 2. Experimental Section

### 2.1. Subjects

There were 25 female subjects recruited from Hacettepe University and the surrounding community. Inclusion criteria included women in good health between the ages of 19–25 who were non-smokers, not dieting to gain or lose weight, not diagnosed with any metabolic diseases, not professional athletes, not taking medications and possessing no food allergies or extreme dislikes for specific foods. Regular breakfast, snack and lunch consumption was an additional inclusion criterion for participants. Subjects were excluded if they scored >9 on a Beck depression scale and had a measured Body Mass Index (BMI) <18 or >30 kg/m^2^. Since menstrual cycle may have an effect on appetite ratings, experiment days were arranged as two weeks before menstruation for all participants. Ethical approval was confirmed on 8 January 2014 by the Non-Interventional Clinical Researches Ethics Board of Hacettepe University (GO14/29-35). Each subject signed an informed consent document before the study.

### 2.2. Study Design

The study took place at the Nutrition Laboratory in the Faculty of Health Sciences, Hacettepe University, Ankara, Turkey. Subjects were tested in a single blind, randomized, crossover design with four products closely matched for energy content. During four different experiment days, a test meal including either milk chocolate-control (CON), oat β-glucan (B-GLU), dark chocolate (DARK) or oat β-glucan and dark chocolate (B-GLU + DARK) was served and, four hours later, an *ad libitum* lunch was offered. A randomization scheme was generated using the website “randomization.com” to allocate participants to the test meals [29]. Each test meal was packed in lettered containers marked A, B, C, D by an investigator who was not involved during trial days. Test meals were served to the corresponding subject according to the randomization letters. Throughout the study, a blinded-trail approach was observed and subject assignment schedules were not seen by either subject or researcher involved in data collection during the trial days.

### 2.3. Test Meals

The test meals used in this study originated from a recipe which includes milk chocolate (40 g), petit beurre biscuits (42 g), hazelnut (~3 g), milk (20 g) and cinnamon (~3 g) (CON). After grinding petit beurre biscuits and hazelnut milk, cinnamon and melted milk chocolate were added to the mixture and then kneaded for portioning. Test meals other than CON were developed with a similar recipe, except that milk chocolate was substituted with dark chocolate (40 g) (DARK) or oat β-glucan (25 g) was replaced with 12 g of petit beurre biscuits (B-GLU) or milk chocolate was substituted with dark chocolate (40 g) and oat β-glucan (25 g) was replaced with 12 g of petit beurre biscuits (B-GLU + DARK). Test meals were covered with coconut powder to provide a similar appearance. Oat β-glucan was purchased from Betaglucare (Prorsum Healthcare, Ytterby, Sweden) which includes 3 g of β-glucan per portion (25 g). The nutritional value of the chocolates were as follows: 100 g milk chocolate energy: 553 kcal, fat: 34.5 g, saturated fatty acids: 19.3 g, carbohydrates: 50.7 g, sugar: 48.0 g, protein: 8.3 g, fiber: 3.4 g; 100 g dark chocolate energy: 487 kcal, fat: 34.1 g, saturated fatty acids: 21.7 g, carbohydrates: 50.9 g, sugar: 31.1 g, protein: 11.6 g, fiber: 17.5 g. Table 1 shows nutritional composition and weight of the test meals.

**Table 1:** Nutritional composition and serving weight of test meals.

Nutrient	Test Meals			
	CON	B-GLU	DARK	B-GLU + DARK
Energy (kcal)	441.05	459.29	414.65	432.89
Carbohydrates (g)	55.22	56.22	60.10	56.30
Sugar (g)	28.44	26.50	23.00	19.74
Protein (g)	6.99	10.41	8.74	11.73
Fat (g)	20.00	19.77	20.38	19.61
Fiber (g)	2.61	8.91	8.40	14.55
β-Glucan (g)	-	3.0	-	3.0
Cocoa (%)	32	32	70	70
Serving (g)	108	121	108	121

Test meals were served together with 250 mL water. The *ad libitum* lunch consisted of pizza, Turkish pastry, fruit juice, yogurt drink and fizzy drinks. Energy and nutrient composition of the food and beverages served at lunch were calculated from the manufacturers’ data.

### 2.4. Experimental Protocol

On experiment days, subjects arrived at department in the morning after fasting for 12 h. Subjects’ body weight, height and body composition were measured (Tanita BC418) and baseline appetite sensations were recorded before eating the test meal. Appetite ratings were recorded on 100 mm visual analog scales (VAS) with words anchored at each end, describing the extremes of a unipolar question (for instance, for hunger: “I am not hungry at all”/“I have never been more hungry”) [30]. All subjects were educated on how to fill in VAS forms. VAS was used to assess hunger, satiety, prospective food consumption, amount of food they could consume, desire for sugary foods and how well the test meal was liked. Subjects were asked how well they liked the test meal at 15 min (immediately after consuming test meal) and other appetite sensations were recorded every 30 min for 4 h. An *ad libitum* lunch was served 4 h after the test meal was consumed. The subjects were instructed to have lunch until comfortably satisfied within 30 min. The same amount and same type of foods were served on the four test days. Energy intake of subjects was measured by weighing the amount of food and drinks consumed and converting this into energy (kcal). Subjects were allowed to sit in the experiment room and read or use laptops throughout the experiment. However, physical activity and social interaction were limited. Subjects were not allowed to see how much other subjects consumed.

### 2.5. Statistical Analyses

Data were analyzed using the Statistical Package for the Social Sciences (SPSS) version 22 (SPSS Inc., Chicago, IL, USA). The primary outcome of this trial was to assess the effect of the test meal on energy intake. The secondary outcome variables were subjects’ VAS scores. For the primary outcome, data was analyzed using a general linear model, ANOVA. Where the ANOVA was significant, Bonferroni *post hoc* analysis was used for comparisons between conditions. VAS data on satiety indicators was analyzed using a repeated measures ANCOVA with baseline measurement as the covariate. Test meal and test meal × time interaction were taken into account. The VAS score for “how well the subjects liked the test meals” and water intake of the subjects were tested by ANOVA. Data was given as mean ± standard error of mean unless otherwise stated. *p* < 0.05 was considered statistically significant. Data on the area under the curve (AUC) for VAS were obtained using GraphPad Prism version 6 (Graphpad Software Inc., La Jolla, CA, USA) and analyzed using ANOVA. Power analysis indicated that a sample of 23 subjects was sufficient to detect a minimum 18.35% energy intake difference with a power of 80% and alpha 0.05 [31].

## 3. Results

All subjects completed the study successfully and all subject data was analyzed for each test meal. The 25 participants were 23.72 ± 1.6 (mean ± S.D.) years of age and weighed 67.91 ± 8.5 kg with a body mass index of 23.20 ± 1.87 kg/m^2^. Although there was a trend towards a difference in how well the subjects liked the test meals, this did not reach to a significant level (CON: 79.78 ± 6.22 mm, B-GLU: 67.76 ± 6.22 mm, DARK: 62.56 ± 6.23 mm and B-GLU + DARK: 66.23 ± 6.22 mm, *p* = 0.096).

**Figure 1:**
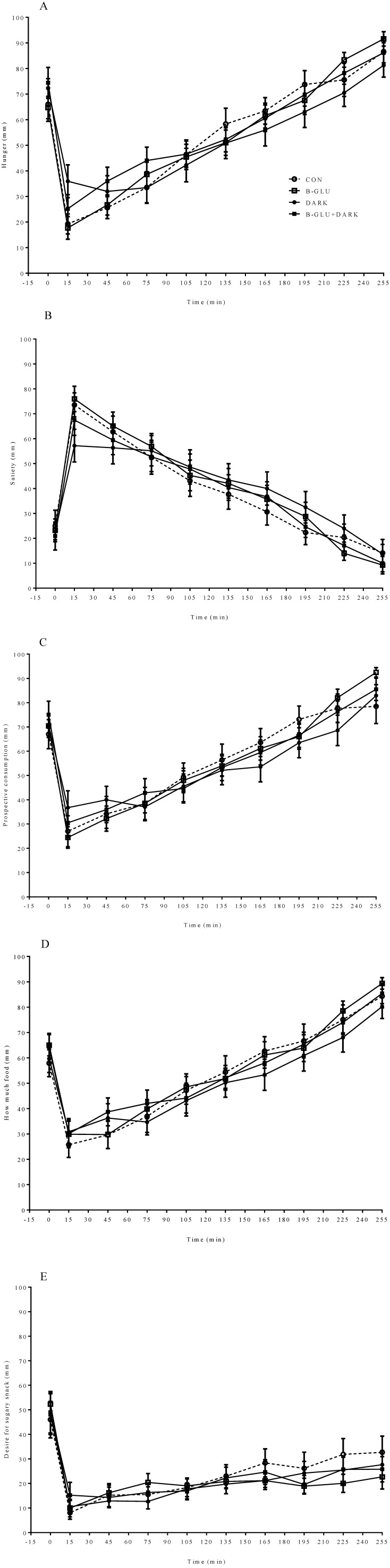
Mean VAS scores during the four test days. Mean VAS scores (±S.E.M.) during the four test days, *n* = 25. (**A**) Hunger; (**B**) satiety; (**C**) prospective food consumption; (**D**) amount of food that could be consumed; (**E**) desire for sugary foods. There were no statistically significant differences between treatments (ANCOVA).

VAS scores did not differ within the four types of test meals for hunger, satiety, prospective food consumption, amount of food that could be consumed and desire for sugary foods (Figure 1). No interaction was detected between test meal and time (Figure 1). In addition, AUC data of VAS scores did not exhibit a significant difference between the groups (Table 2). There was no difference in water intake during the four test days (CON: 231 ± 12 mL, B-GLU: 220 ± 16 mL, DARK: 238 ± 9 mL and B-GLU + DARK: 224 ± 11 mL, *p* > 0.05).

**Table 2:** AUC data of VAS scores.

VAS Questions	Test Meals				
	CON	B-GLU	DARK	B-GLU + DARK	*p* Value
Hunger	469.93 ± 37.19	470.13 ± 35.24	460.71 ± 38.32	482.51 ± 37.51	0.94
Satiety	367.03 ± 38.38	379.84 ± 36.28	375.34 ± 40.16	368.65 ± 34.52	0.99
Prospective consumption	488.72 ± 40.21	485.84 ± 45.23	473.89 ± 39.54	487.88 ± 38.21	0.99
Amount of food that could be consumed	469.43 ± 38.34	482.21 ± 37.34	447.44 ± 38.28	479.88 ± 36.87	0.87
Desire for sugary snack	203.77 ± 30.32	183.85 ± 30.56	182.33 ± 29.65	185.75 ± 33.02	0.96

**Figure 2:**
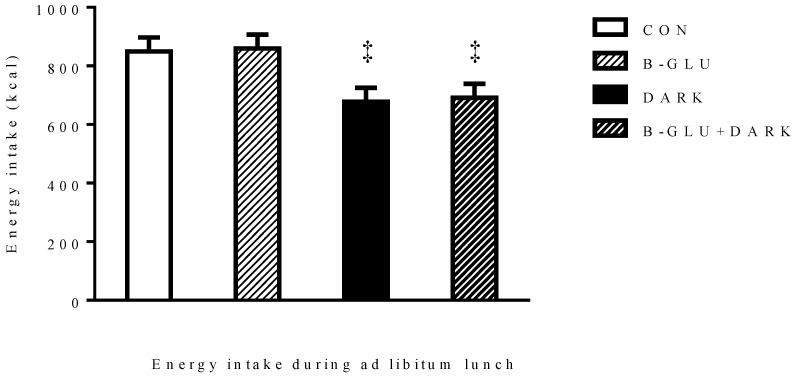
Mean energy intakes during *ad libitum* lunch.

Despite no difference in VAS scores of appetite ratings, when DARK and B-GLU + DARK were compared to CON energy intake, the following *ad libitum* meal was 20.2% and 18.64% lower in DARK and B-GLU + DARK test meals, respectively (CON: 849.46 ± 47.45 kcal, DARK: 677.69 ± 48.45 kcal and B-GLU + DARK: 691.08 ± 47.45 kcal, *p* = 0.014) (Figure 2). Adjusting for how well the subjects liked the test meals did not alter significance. B-GLU group did not exhibit a significant difference in energy intake with respect to CON (Figure 2). No significant difference was found between DARK and B-GLU + DARK test meals (Figure 2). Thus, switching from milk chocolate to dark chocolate in test meals resulted in a reduced energy intake during the following meal. However, no effect of added β-glucan was found during the open buffet lunch.

Mean energy intakes (±S.E.M.) during *ad libitum* lunch, *n* = 25; ‡ indicates that subjects had lower energy intake in DARK and β-GLU + DARK groups (*p* = 0.014).

## 4. Discussion

The current study compared the satiating effects of four different products made from the same ingredients but modified according to their β-glucan content and chocolate type. The VAS scores at specific time points did not indicate a significant effect of different test meals on appetite sensing. However, when the energy intake of individuals during the *ad libitum* lunch were measured, the results showed that only dark chocolate was associated with lower energy intake. Therefore, β-glucan did not exert an influence on any of the appetite ratings and food intake in the current study. The crossover design of the study favored the minimum variation in participant energy consumption and perception. This is the first study comparing the satiating effects of the combined functional food components of β-glucan and dark chocolate.

The dark chocolate used in this study contained 70% cocoa, which can be considered at the higher end of a dense flavoring scale. The main reason for choosing this type of chocolate was to compare the effect of a strong dark chocolate with a regular milk chocolate; both chocolates were substituted gram for gram within the different recipes in order to study the comparative nutritional effects involved in this nutritional alteration. Since the nutritional value of the chocolates were different despite using the same amounts, a slightly different nutritional composition in test meals was produced. It is clear that the significant change in energy intake of individuals can be explained by these differences although this makes the interpretation and discussion of the results more complex.

One of the most important points is that the dark chocolate used in this study contains lower energy than milk chocolate and this was reflected in total energy of the test meals. In a previous study showing that the dark chocolate promoted increased satiety when compared to milk chocolate, dark chocolate had higher calorie content [24]. It was argued that the difference in satiety levels cannot be attributable to the higher energy content of dark chocolate since results remained significantly higher when adjusted for extra energy in that study. Our results support this notion, as the DARK group resulted in lower energy intake during *ad libitum* lunch despite its slightly lower energy content. Furthermore, the protein content of the test meals also varied according to their dark chocolate and oat content. Recent meta-analysis regarding the effects of high protein meals on satiety revealed that acute consumption of high protein resulted in a pronounced satiety [32]. However, in the current study, B-GLU had a higher content of protein when compared to DARK and did not induce reduced energy intake during the lunch. Therefore, the difference in protein content of the test meals in this study did not exert an impact upon satiety response.

The influence of sweet taste on short-term satiety and energy intake has been shown previously [33,34]. According to this evidence, habitual consumption of sweet products can affect appetite as a consequence of a relationship between sweet taste and energy. The subjects in the current study were regular chocolate consumers and the test meals contained different amounts of sugar. Due to the fact that inclusion of dark chocolate and oat β-glucan to the recipe resulted in a lower content of sugar, the B-GLU + DARK group had the lowest amount of sugar. Thus, the differences in energy intake during the *ad libitum* lunch can be attributable to the sugar content of the test meals. However, there was still a 14.17% difference between the sugar content of DARK and B-GLU + DARK and this difference did not lead to a significant difference between these groups. Measurement of the sweetness of the test meals along with related physiological parameters should be the next step for future studies.

A further potential satiating effect of dark chocolate may be due to its cocoa content which leads distinctions in flavor intensity. Previously, it was shown that the intensity of foods was related with sensory specific satiety [25]. Since the cocoa origin and composition of dark chocolate were also found to be effective on consumer appetite [35], the intense flavoring of 70% cocoa dark chocolate may have also led to a decreased food intake. Although fat content of the dietary components was reported to be a deterministic factor in regulation of appetite [36], the fat content of the test meals were similar in this study. However, the composition of fat in two chocolate types was distinctly different as dark chocolate mainly includes cocoa butter. Cocoa butter contains a high proportion of stearic acid which was reported to be less digestible and bioavailable than other fatty acids [37]. However, a randomized study in humans revealed that cocoa butter in dark chocolate had similar digestibility to corn oil, thereby indicating that cocoa butter was also well absorbed [38]. At this point, determination of gastro-intestinal transit time of cocoa butter gains more importance since delay in transit time may exert different effects on absorption and satiety-related factors. In addition to fatty acid composition, the polyphenol content of cocoa can also be considered as an important factor [39]. There is evidence showing the positive effects of having cocoa flavanols as part of a diet on oxidative status [40] and glucose metabolism [23] and, as the current study indicates, although cocoa polyphenols may also play a role in conducting satiety responses, this hypothesis needs to be corroborated by future studies.

Dietary fiber is an important component of a healthy and well-balanced diet. To date, several studies indicated the beneficial effects of fiber intake on body composition, energy intake and appetite regulation [41,42,43]. It is well-established that dietary fiber may enhance satiety through various mechanisms such as delaying gastric emptying, slowing glycemic response and increasing gastric distension [44,45]. In addition to the factors that are associated with the satiating effects of dark chocolate, the fiber content of cocoa can also be responsible for the reduced energy intake since cocoa fiber was shown to be linked with several health benefits [46,47]. Moreover, oat β-glucan used in this study provided 3 g of β-glucan, which was considered to be effective in promoting acute term satiety [48,49,50]. However, we did not observe a significant effect of β-glucan on short-term energy intake. In fact, our results showed consistent outcomes with other studies that examined the effects of β-glucan-enriched or supplemented products on short-term satiety [51,52,53,54], excluding a study of using 8 g of β-glucan in biscuits and juice [55]. It was reported that molecular weight and solubility of β-glucan were important factors that determine the physiologic effect [56,57]. Although the high content of β-glucan in test meals was expected to exert enhanced viscosity and thus increased satiety, molecular weight or solubility of the β-glucan might have influenced outcomes. This may explain the observed results partly since the amount of extractable β-glucan was shown to produce a greater effect than the total amount of β-glucan in a previous study [58]. In addition, a recent systematic review revealed that short-term fiber treatments did not impact upon food intake and factors such as fiber type or fiber dose and they were not associated with satiety responses [54]. It appears that studies which compare whole grain foods rather than additive forms of dietary fiber to high glycemic index foods or foods containing no dietary fiber have illustrated improved appetite control and increased satiety [59].

The current study consisted of a population of healthy women. Although the effect of menstrual cycle was controlled in order to eliminate the possible effects of menstrual cycle on appetite [60,61], one study showed that chocolate tasting in men and women created different responses to satiety [62]. Therefore, the present study should be repeated in men and more analyses should be performed in order to elucidate the related mechanisms behind the differences. Furthermore, palatability is an important factor in determining satiety [63]. The current study did not measure palatability response on test foods. Nevertheless, after the consumption of test meals, a question was added into the VAS questionnaire about how well the participants liked the test food; results exhibited that, despite a statistical trend, liking of the test meals were similar and none of the participants mentioned that the test foods were unpleasant. In addition, satiety and appetite responses were measured through subjective ratings of 100 mm VAS questionnaires. Due to the fact that dietary fiber and fiber-related factors are closely connected to a series of physiological responses including insulin secretion, satiety-related hormones and gut peptides, it is crucial to measure these objective parameters in future studies.

## 5. Conclusions

In the current study, consumption of a dark chocolate-based snack reduced hunger more than the milk chocolate-based recipe. More physiological studies are needed to analyze the mechanisms of the beneficial effects of β-glucan and dark chocolate supplementation and/or substitution. The data obtained from this study suggests that individuals switching from milk chocolate to dark chocolate would decrease their energy intake in the short term.
